# ‘’Wait and See’’ as a Treatment Option for a Rathke’s Cleft Cyst Apoplexy in Pediatric Population: A Case Report

**DOI:** 10.5812/ijem-143939

**Published:** 2024-03-16

**Authors:** Siham Rouf, Latifa Yaqoubi, Hanane Latrech

**Affiliations:** 1 Department of Endocrinology Diabetology and Nutrition, Mohammed VI University Hospital, Faculty of Medicine and Pharmacy of Oujda, Mohamed the First University, Oujda, Morocco; 2 Laboratory of Epidemiology, Clinical Research and Public Health, Faculty of Medicine and Pharmacy of Oujda, Mohammed the First University, Oujda, Morocco

**Keywords:** Rathke Cleft Cyst, Apoplexy, Conservative Treatment, GH Deficiency

## Abstract

**Introduction:**

Rathke cleft cyst apoplexy is exceedingly rare, particularly in infants. The most prevalent clinical manifestations include headaches, visual field defects, and endocrine dysfunction. Treatment options range from surgery to conservative methods, taking into consideration the balance of benefits and risks, especially during critical life stages such as childhood.

**Case Presentation:**

We present the case of a 12-year-old boy admitted due to the recent onset of headaches and diabetes insipidus. Magnetic resonance imaging revealed Rathke cleft cyst apoplexy. Given the absence of compressive symptoms in a child at the early stages of puberty and without abnormalities in basic endocrine tests, a conservative strategy was employed, involving regular clinical, biological, and radiological follow-ups. The child experienced normal puberty without any endocrine deficiencies except for a partial growth hormone deficiency.

**Conclusions:**

For clinically asymptomatic children diagnosed with Rathke's cleft cyst apoplexy, adopting a conservative management approach is recommended, provided there is thorough clinical, biological, and radiological surveillance.

## 1. Introduction

Rathke's cleft cysts (RCCs) are benign sellar-suprasellar cysts that originate from epithelial remnants of the Rathke pouch, forming around the third or fourth week of embryonic development (1). These cysts are typically asymptomatic and often incidentally identified through brain magnetic resonance imaging, with a prevalence of 3.9% in adults and 1.2% in children under 15 (2). Apoplexy represents a rare complication of RCC, particularly in the pediatric population, with a limited number of cases reported (3, 4). Surgery is considered the primary treatment for symptomatic individuals experiencing acute headaches, visual field defects, or oculomotor palsies. Nonetheless, for patients without symptoms of mass effect, a conservative strategy involving regular monitoring and hormonal replacement may be considered (5).

We detail the case of a 12-year-old boy admitted for diabetes insipidus (DI), in whom RCC apoplexy was identified on MRI. Notably, this is the first reported instance of RCC apoplexy in a young boy managed conservatively. The clinical, biological, and radiological findings were examined. The follow-up process, which follows a conservative management approach, is also discussed.

## 2. Case Presentation

A 12-year-old boy was brought to our department exhibiting sudden onset symptoms of headache and polyuria-polydipsia syndrome, which began one week prior to his initial visit. The child had no significant medical history. During the first clinical evaluation, he measured 146.5 cm in height (M) and weighed 30 kg (-1.4 SD). There were no observed signs of adrenal insufficiency or hypothyroidism. He was at the onset of puberty, with gonad sizes measuring 3.2 cm on each side and a penis length of 6.2 cm (M). Notably, the patient experienced polyuria-polydipsia syndrome, with fluid excretion reaching up to 113ml/kg/day, nocturnal enuresis, and an excessive liquid intake of 3.8 liters/m². Ophthalmologic examination yielded expected results, with no visual impairments detected and normal optical coherence tomography (OCT) findings.

The biological assessment revealed DI, with a serum sodium level of 140 mEq/l and plasma osmolality of 287 mosm/kg, while the urine osmolality was significantly low at 179 mosm/kg. Furthermore, his serum levels of insulin-like growth factor-1 (IGF1), prolactin (PRL), free T4, cortisol, follicle-stimulating hormone (FSH), and luteinizing hormone (LH) were all within the normal range (Table 1). 

**Table 1.** Clinical Characteristics and Biological Assessment at the Baseline and During the Follow-Up

Variables	At Baseline	After 1 Year	After 2 Years	After 3 Years
**Height**	146.5 (M)	147 (M)	150 (-0.8 SD)	156 (-1.1 SD)
**Tanner stages**	Tanner II	Tanner III	Tanner IV	Tanner V
**IGF1, ng/ml ** ^**a**^	118.2	-	-	183.5
**Prolactin, ng/ml (Norms: 3.4 - 19.4)**	13.9	32	17.7	13.1
**Cortisol, ng/ml (Norms: 37 - 194)**	137	171.8	156.7	126
**Free T4, pmol/l (Norms: 10.6 - 19.4 )**	12.6	12.0	9.3	13.6
**FSH, mUI/l (Norms: 1.5 - 14)**	1.7	1.47	3.03	3.82
**LH, mUI/l (Norms: 1.2 - 10)**	0.6	0.78	2.4	3.52
**Testosterone, ng/ml ** ^**b**^	0.12	0.12	6.8	5.7

Abbreviations: IGF1, insulin-like growth factor-1; PRL, prolactin; FSH, follicle-stimulating hormone; LH, luteinizing hormone.

^a^ IGF1 (Chemiluminescence, The norms interpreted depending on puberty stages) (16).

^b^ The norms of testosterone vary depending on puberty stages: Tanner I (0.02 - 0.3), Tanner II (0.03 - 2.8), Tanner III (0.08 - 6.8), Tanner IV (0.17 - 7.8), Tanner V (0.13 - 9.06).

MRI scans with and without contrast highlighted apoplexy in an RCC, showing a spontaneous hyperintensity on T1 and T2 sequences measuring 15x6x11 mm. The anterior pituitary gland displayed homogeneous contrast uptake. However, we observed a loss of the typical hyperintensity of the posterior pituitary gland, with no radiological indications of a craniopharyngioma. Therefore, during the initial hormonal evaluation, the only hormone deficiency identified in our case was DI, which showed significant improvement under vasopressin treatment. The case was reviewed in a multidisciplinary meeting, including an endocrinologist, neurosurgeon, and radiologist. Given the absence of clinical or biological signs other than DI and the stability of the RCC apoplexy over nine months of MRI monitoring—with measurements of 12 × 11 × 10 mm—a conservative management approach with regular follow-ups was chosen.

**Figure 1.**
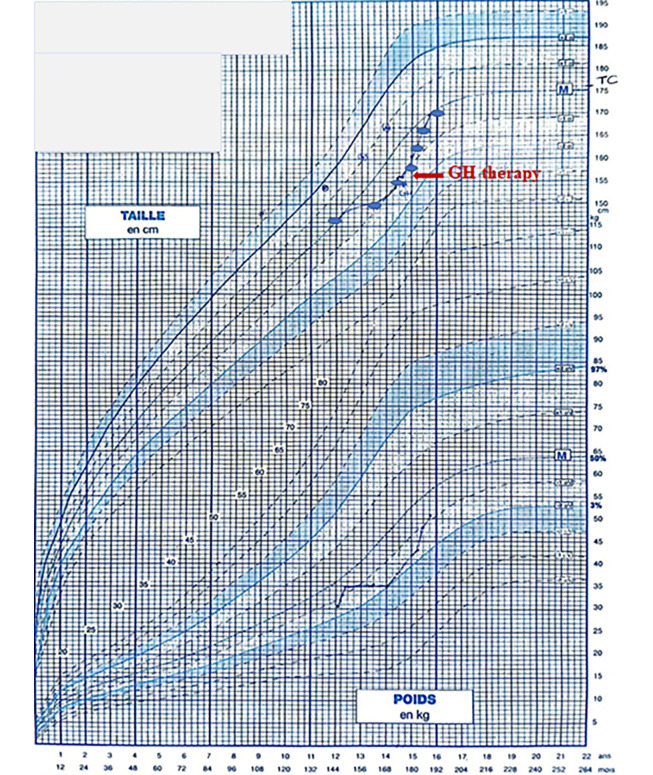
Growth chart showing the decline of growth velocity and the gain after GH therapy

The child was monitored for three years, during which he displayed normal puberty development and maintained a stable hormonal profile (Table 1). Visual evaluations revealed no abnormalities. However, a decrease in growth velocity was noted, dropping from -0.8 SD to -1.1 SD. This necessitated an investigation for acquired growth hormone (GH) deficiency. At the age of 14 years and nine months, the child was readmitted to undergo two GH stimulation tests: A propranolol-glucagon test and an Insulin Tolerance Test, which indicated a partial GH deficiency with peaks of 19.3 µUI/ml and 10.0 µUI/ml, respectively. After receiving GH treatment, the patient experienced a notable increase in growth velocity, improving from -1.1 SD to 0.2 SD over one year (Figure 1). 

**Figure 2.**
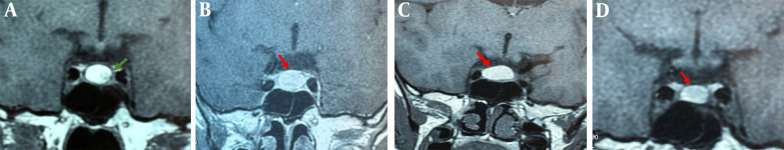
Pituitary MRI of our patient showing the evolution of the RCC apoplexy at the baseline (A), after 9 months (B), 2 years (C), and 3 years (D) after a conservative approach

Subsequently, MRI monitoring showed a stable appearance of the RCC apoplexy after two years (11 × 12 × 11 mm), with a slight decrease in size observed at the three-year follow-up, measuring 7 × 10 × 6 mm (Figure 2). It is important to note that the radiological follow-up was consistently conducted at the same MRI center, with the same radiologist interpreting both recent and previous MRI findings during multidisciplinary meetings that included an endocrinologist and a neurosurgeon.

## 3. Discussion 

RCC apoplexy is an exceptionally rare occurrence, particularly within the pediatric demographic, with very few cases documented in the literature (3, 4). Although several theories have been proposed, the precise mechanism behind this complication remains uncertain. One theory suggests the overproduction of mucopolysaccharides, leading to slight compression and intracystic hemorrhage (6). Other theories focus on the cyst wall's fragility and the potential for repeated minor hemorrhages from bleeding vessels (3, 7).

However, no iatrogenic factors such as trauma, cardiovascular interventions, radiation, or anticoagulation therapy have been identified as contributing to the apoplexy of RCC (7).

Pediatric RCC apoplexy is exceedingly rare. Patients with RCC apoplexy present with clinical manifestations similar to those with pituitary tumor apoplexy, including almost invariably sudden headaches, which may be accompanied by nausea, cranial nerve palsies, visual field defects, or hormonal dysfunction (8).

The first reported case of pediatric RCC apoplexy was described by Kurisaka et al., involving an eight-year-old girl who experienced a sudden onset of headache and deep ophthalmic pain without any visual impairments. Her visual acuity and Goldman's visual field tests were normal, as were her mental and physical evaluations, showing no endocrine abnormalities (3). Surgery was deemed necessary to prevent any further apoplectic episodes, resulting in significant clinical improvement post-operation. Martinez Santos et al. documented the cases of two teenage sisters: The first, a 13-year-old girl, was admitted with sudden headaches, nausea, and a peripheral vision defect. Neurological evaluation revealed incomplete hemianopsia, although her visual acuity remained normal. The presence of neuro-ophthalmological disturbances warranted transsphenoidal surgery, which immediately resolved her visual field defect (4). The second sister, aged 17, had a lengthy history of generalized tonic-clonic seizures and abnormal electroencephalography findings. The recent onset of headaches led to an MRI scan, which identified RCC apoplexy. The acute symptoms combined with radiological evidence of cyst enlargement necessitated endonasal endoscopic transsphenoidal decompression. Post-surgery, her seizures significantly decreased in frequency (Table 2) (4).

**Table 2.** Summary of RCC Apoplexy Pediatrics Cases from the Literature

References	Age/Sex	Clinical Presentation	Hormonal Deficits	MRI	Management	Short-Term Outcome	Long-Term Outcome
**Kurisaka et al. (1998) (** 3 **)**	8 years/ female	Headache; vision pain	None	NA ^a^	Trans-sphenoidal surgery	Improved	NA
**Martinez Santos et al. (2019) (** 4 **)**	13 years/female	Headache; visual defects; hemianopsia	None	NA	Trans-sphenoidal surgery	improved	NA
**Martinez Santos et al. (2019) (** 4 **)**	17 years/female	Headache; seizure history	None	7 mm	Trans-sphenoidal surgery	Improved	Less frequency of seizures
**Rouf et al. (2023) **	12 years/male	Headache; diabetes insipidus	None	12 × 11 × 10 mm.	Conservative	Improved	Normal puberty: The onset of partial GH deficiency

^a^ The information was not mentioned in the article.

The primary symptoms observed in our case were headache and sudden onset of DI, with the latter being a distinctive feature among all reported pediatric cases of RCC apoplexy (3, 4). We theorize that the acute symptoms might arise from a compressive syndrome that causes local irritation of surrounding structures, coupled with aggressive intracystic production of mucopolysaccharides and local hemorrhage (4). Oishi et al. documented cases where acute headache paired with DI in a 63-year-old female and an 8-year-old boy with RCC was histologically confirmed by the presence of infiltration by numerous inflammatory cells in both the anterior and posterior pituitary lobes, attributed to panhypophysitis caused by minor ruptures of RCCs (9)).

Surgery is recommended for patients presenting acute pressure signs, especially optic nerve compression, with the endonasal endoscopic transsphenoidal route being preferred. This surgical approach involves opening the floor of the Sella Turcica, allowing for the drainage of a mixture of hemorrhagic fluid, mucinous material, and pituitary gland tissue. It is possible to evacuate RCC cysts without necessitating their complete removal (10, 11). Postoperative outcomes have shown significant clinical improvement in patients (3). However, it has been noted that patients with preoperative hormonal deficiencies may require long-term hormone replacement therapy (12).

Besides headache and DI, our patient was asymptomatic, and all initial biological tests returned normal results. After a thorough discussion in a multidisciplinary meeting, a conservative management strategy was chosen, involving regular clinical, biological, and radiological monitoring. The child demonstrated consistent growth velocity and normal puberty development over two years. However, during the last year of follow-up, we observed a reduction in his growth velocity, indicating the recent onset of GH deficiency.

When patients are asymptomatic with normal hormonal evaluations, a conservative approach may be suitable, focusing on regular clinical, hormonal, and radiological monitoring. This strategy entails careful monitoring for symptoms of hormonal deficiencies, conducting biological assessments, and tracking the cyst's size through regular MRI examinations. Surgery has been the indicated treatment in previously reported cases of RCC apoplexy that presented with neurological and ophthalmological signs. Patients who required surgical intervention exhibited visual impairments, seizures, and episodes of epilepsy (3, 4). When following a conservative approach with asymptomatic patients, clinicians must promptly remain alert to detect any signs of pituitary dysfunction, ensuring that surgical intervention is administered when necessary.

We acknowledge that a significant limitation of this report is the lack of a histological diagnosis, given that surgical intervention is considered the standard treatment for these cases. Nonetheless, we opted for an initial conservative approach for an asymptomatic child who exhibited no visual field disturbances or hormonal dysfunctions except for DI. Additionally, our patient experienced an average onset of puberty, which developed perfectly over the three years of follow-up. We assessed that the likelihood of endocrine function recovery was minimal. The literature review reveals mixed outcomes regarding endocrine improvement post-surgery. A study involving 28 symptomatic RCC patients showed that only 15% of those with panhypopituitarism exhibited improvement following surgery (13). Another review encompassing 29 RCC patients found that the hemorrhagic type was predominant (86%), with over half of these patients experiencing hormonal deficits (52%). Postoperatively, 45% of patients required hormonal replacement (14). Furthermore, there have been instances where patients developed new cases of DI following surgery (15). Oishi et al. also reported cases of persistent DI post-surgery, albeit controlled with a reduced dose of desmopressin (9).

RCC apoplexy may manifest suddenly with symptoms such as headaches, visual field deficits, and hormonal dysfunction. Managing this rare condition in the pediatric population poses significant challenges. The therapeutic approach varies between surgery and conservative management, aiming primarily to preserve average growth and puberty development in clinically asymptomatic children. To our knowledge, this is the first reported case of conservative management of pediatric RCC apoplexy without neurological and visual impairments. However, a long-term follow-up remains crucial.

## Data Availability

Further data are available from the corresponding author upon reasonable request.
